# Anti-staphylococcal fatty acids: mode of action, bacterial resistance and implications for therapeutic application

**DOI:** 10.1099/mic.0.001563

**Published:** 2025-05-22

**Authors:** Edward J.A. Douglas, Nathanael Palk, Emily R. Rudolph, Maisem Laabei

**Affiliations:** 1 Centre for Bacterial Resistance Biology, Imperial College London, London W2 1NY, UK; 2 School of Cellular and Molecular Medicine, University of Bristol, Bristol BS8 1TD, UK; 3 Department of Life Sciences, University of Bath, Bath BA2 7AY, UK

**Keywords:** fatty acids, mode of action, resistance mechanisms, *Staphylococcus aureus*, therapeutics

## Abstract

Novel strategies to counter multidrug-resistant pathogens such as methicillin-resistant *Staphylococcus aureus* are urgently required. The antimicrobial properties of fatty acids (FAs) have long been recognized and offer significant promise as viable alternatives to, or potentiators of, conventional antibiotics. In this review, we examine the interplay between FAs and *S. aureus*, specifically detailing the underlying molecular mechanisms responsible for FA-mediated inhibition and the counteracting staphylococcal systems evolved to withstand FA onslaught. Finally, we present an update on the recent therapeutic FA applications to combat *S. aureus* infection, either as a monotherapy or in combination with antibiotics or host-derived antimicrobial peptides. Given the frequency of interaction between FAs and *S. aureus* during host colonization and infection, understanding FA mode of action and deciphering *S. aureus* FA resistance strategies are central in rationally designing future anti-staphylococcal FAs and FA-combination therapies.

## Introduction

Fatty acids (FAs) are comprised of a hydrophilic carboxyl head group and a hydrophobic carbon tail. They can be grouped based on the hydrocarbon chain’s length or degree of saturation. Based on their length, FAs are organized as short-chain FAs (SCFAs) (less than 6 carbons), medium-chain FAs (between 6 and 11 carbons) and long-chain FAs (LCFAs) (more than 11 carbons). According to their degree of saturation (or the number of double bonds), FAs can be classified as saturated (containing only single bonds), monounsaturated or polyunsaturated (PUFA) FAs, the latter two containing one or more double bonds, respectively [1]. FAs are employed as innate immune defence agents by diverse eukaryotic organisms including plants, algae and mammals [2]. FAs are found scattered throughout the human body and can be isolated from most tissues; however, hot spots, with the highest FA biosynthesis, occur in the liver and adipocytes [3]. Host-specific FAs such as sapienic acid (*cis*-6-hexadecenoic acid), exclusively produced by humans [4], are important colonization barriers that can restrict or eliminate several bacterial pathogens, including the opportunistic, multidrug-resistant (MDR) human pathogen, *Staphylococcus aureus*. Humans [5] and mice [6] deficient in the production of sapienic acid are more susceptible to *S. aureus* skin infections. Moreover, patients suffering from atopic dermatitis (AD), a chronic skin disease frequently exacerbated by *S. aureus*, display decreased levels of both sapienic acid and oleic acid, suggesting that deficiency in FAs contributes to staphylococcal skin pathophysiology [7].

*S. aureus* is a major human pathogen that can cause a myriad of illnesses ranging from superficial skin diseases to life-threatening systemic infections and toxinoses [8].

Treatment of *S. aureus* infections has become complicated by the global emergence of multiple lineages that have gained resistance to clinically relevant antibiotics [9]. Recent predictive statistical models estimate that methicillin-resistant *S. aureus* caused more than 100,000 deaths worldwide in 2019 [10]. Furthermore, studies investigating global antimicrobial resistance burden across the 1990–2021 timespan have concluded that deaths associated with and attributable to methicillin-resistant *S. aureus* (MRSA) have increased the most from a panel of 22 pathogens and 84 pathogen-drug combinations [11]. Accordingly, the WHO recognizes *S. aureus* as a priority pathogen where there is a critical need for the development of new antibiotics and immunotherapeutics to combat *S. aureus* diseases [12].

A suite of FAs has been observed to possess anti-staphylococcal activity (Table 1); for simplicity, we refer to FAs by both their ‘organism identifier’ and the delta system nomenclature. Renewed interest in exploring the use of FAs or FA-associated therapies to combat *S. aureus* infection has grown in recent years, motivated by the rise of antimicrobial-resistant *S. aureus* and the accompanying increase in morbidity and mortality associated with *S. aureus* disease globally [13].

**Table 1.** FAs with reported toxicity to *S. aureus*

Name	Delta naming system	Anti-staphylococcal activity
***Saturated FAs***		
Caprylic acid	C8:0	[195,196]
Capric acid	C10:0	[56,196, 197]
Undecanoic acid	C11:0	[56,193, 198]
Lauric acid	C12:0	[4,65, 193, 196, 197]
Myristic acid	C14:0	[65,196, 197, 199]
***Unsaturated FAs***		
Myristoleic acid	C14:1Δ9	[56,193, 197, 200]
Sapienic (*cis*-6-hexadecenoic acid) acid	C16:1Δ6	[4,21, 22, 65, 145, 196]
Palmitoleic acid	C16:1Δ9	[4,65, 121, 197]
Vaccenic acid	C18:1Δ7	[56]
Oleic acid	C18:1Δ9	[56,67, 168, 169]
Petroselinic acid	C18:1Δ12	[(56]
Linoleic acid	C18:2Δ9, Δ12	[60,65, 66, 201]
*α*-Linolenic acid	C18:3Δ9,12,15	(65,201]
*γ*-Linolenic acid	C18:3Δ6,9,12	[65,202]
Arachidonic acid	C20:4Δ5,8,11,14	[56,61, 65, 95]
Eicosapentaenoic acid (EPA)	C20:5Δ5,8,11,14,17	[55,203, 204]
Docosahexaenoic acid (DHA)	C22:6Δ4,7,10,13,16	[55,203]

Accordingly, understanding how FAs function to eradicate bacteria and conversely how bacteria withstand FA onslaught is paramount for the successful use of FAs in combatting bacterial infection, either alone or in conjunction with other therapeutics. This review documents the role of FAs in the context of *S. aureus* colonization and infection and discusses mechanisms of activity of key anti-staphylococcal FAs and the resistance strategies *S. aureus* has evolved to prevent FA-mediated eradication and lastly presents an overview of recent therapeutic applications of FAs to treat *S. aureus* disease.

## Interactions between FAs and *S. aureus*

### Skin

*S. aureus* colonizes ~20–30% of the population in sites such as the nasal mucosa, intestine, groin and axillae [14]. Colonization is harmless but also a recognized risk factor for subsequent disease with up to 80% of *S. aureus* infections originating from the colonizing strain [15,16].

Numerous FAs are found in the nasal mucosa and on the skin (Table 1). On the skin, they are present in high quantities in human sebum, produced from sebaceous glands [4,17]. In combination with a low pH and the presence of antimicrobial peptides (AMPs), FAs protect the skin from colonization of pathogenic bacterial species [18]. Skin microbiota studies have indicated that *S. aureus* colonization is reduced in areas where FAs are found in high amounts [19,20]. A potent anti-staphylococcal FA, sapienic acid, displays rapid bactericidal activity *in vitro* [4,21], and short-term topical application of sapienic acid was associated with a reduction in *S. aureus* recovery from 6/8 AD patients [5].

Skin FAs also play a role as effective anti-virulence agents and potentiators of innate immune responses. Glycerol monolaurate (GML) and lauric acid block the expression of *α*-haemolysin, protein A and toxic shock syndrome toxin, whereas sapienic acid exposure inhibits the transcription of protein A and *α*-haemolysin [22,23]. Unsaturated FAs (uFAs) such as palmitoleic and oleic acid can become incorporated into the lipid moiety of staphylococcal lipoproteins and potentiate the activation of various immune cells in a TLR2-dependent manner [24]. Incubation of human sebocytes with lauric acid, palmitic acid or oleic acid increases the expression of hBD-2, a highly active anti-staphylococcal AMP [25].

Despite the presence of FAs on the skin and in sebaceous glands, *S. aureus* characteristically infects the skin in the form of a carbuncle or furuncle [26]. These infections typically start in a hair follicle [26]. Sebaceous glands are located almost exclusively within or next to hair follicles and together form a pilosebaceous unit [27]. Therefore, *S. aureus* skin infections become established within a niche, which has high concentrations of FAs [4]. This suggests a remarkably high adaptive response to FAs present on the skin. In support of this, a study investigating the virulence gene response of *S. aureus* in a cutaneous infection model using human skin explants found several genes important for FA resistance to be upregulated [28]. In the early stages of infection (2 h), *essA* and *esaA* were upregulated ~2-fold [28]. These genes produce effectors of the type 7 secretion system (T7SS), an apparatus that has been implicated in FA resistance (discussed below). Twenty-four hours after the establishment of infection, the lipase *geh* as well as the surface protein *isdA* was also upregulated [28]. Finally, between 24 and 72 h, the surface protein *sasF*, as well as secreted components of the T7SS*,* was upregulated between 3- and 6-fold [28]. Another study has shown that the production of Geh lipases plays an important role in the promotion of skin inflammation and damage [29]. Furthermore, the authors showed, using a Δlipase strain, that lipases are integral for the ability of *S. aureus* to penetrate the lipid-rich infundibulum of the hair follicle [29]. Taken together, these studies suggest that overcoming the antimicrobial activity of FAs is key to the establishment of infection.

### Bloodstream

Bacteraemia is among the most severe infections caused by *S. aureus* with a high mortality rate of 20–30% [30,31]. Human serum contains several FAs with anti-staphylococcal activity (Table 1), with arachidonic acid being the most abundant [32,33]. In addition, FAs are stored in an esterified form within the host membrane of macrophages [34]. Upon phagocytosis of a pathogen, phospholipase A2 enzymes release free FAs which promote local antimicrobial activity or where they can be oxidized into biologically active lipid mediators, known as eicosanoids [35]. In general, omega-6 FAs, such as arachidonic acid, are oxidized into pro-inflammatory eicosanoids, such as prostaglandins, leukotrienes and thromboxanes, by cyclooxygenase (COX), lipoxygenase and cytochrome P450 enzymes, or via free radical oxidation [36,37]. Accordingly, serum levels of prostaglandins, such as PGE2 and PGD2, are significantly increased in patients with bacterial sepsis compared with healthy controls, and these patients also exhibit increased PLA2 and COX-2 activity [37]. In contrast, omega-3 FAs, such as eicosapentaenoic and docosahexaenoic, are mostly metabolized into anti-inflammatory molecules, such as resolvins, protectins and maresins [38].

Levels of omega-6 and omega-3 FAs and their metabolites are important factors in the outcome of *S. aureus* bacteraemia infections. For example, a decreased level of arachidonic acid, eicosapentaenoic acid, docosahexaenoic acid and linolenic acid in serum was identified as a significant risk factor for poor outcome in a study of 200 sepsis patients [39]. Furthermore, Svahn *et al*. demonstrated that mice fed a polyunsaturated high-fat diet (HFD-P) have a decreased bacterial load and increased survival from an *S. aureus* bacteraemia infection compared with mice fed a saturated high-fat diet (HFD-S) [40]. Interestingly, mice fed HFD-P had a higher frequency of neutrophils in the bone marrow and blood and exhibited higher neutrophil recruitment. A follow-up study demonstrated that specifically dietary omega-3 FAs are responsible for increased survival from *S. aureus* bacteraemia and treatment of mice fed HFD-S with resolvins, a metabolite of omega-3 FAs, decreased bacterial loads in the kidney and improved survival up to 8 days after treatment [41].

### Intracellular environment

*S. aureus* is capable of invading and surviving inside many cell types, including epithelial and endothelial cells, keratinocytes, fibroblasts and osteoblasts [42,43]. Moreover, *S. aureus* can survive and replicate within professional phagocytes [44,45]. The intracellular environment can provide protection from the host immune system and antibiotics, which are unable to penetrate the host cell membrane. Furthermore, intracellular *S. aureus* acts as a reservoir facilitating persistence and dissemination to new sites of infection [43,45,50]. Using untargeted MS-MS, Yan *et al*. found that linoleic acid metabolism was upregulated in macrophage cells infected with *S. aureus* [51]. Furthermore, treatment of *S. aureus*-infected macrophages with linoleic or arachidonic acid significantly reduced intracellular bacterial numbers. Using fluorescent probes, the authors confirmed that linoleic acid induced the production of superoxide and reactive oxygen species (ROS) intracellularly, resulting in bacterial elimination. Finally, the authors demonstrated that multiple treatments of linoleic could decrease bacterial loads in the lung, liver and spleen during an *S. aureus* bacteraemia mouse model [51]. Collectively, the results indicate that FAs are important in the eradication of intracellular bacteria and may have promise as a new therapeutic approach to tackle persistent *S. aureus* infections.

### Biofilm

Biofilms are organized populations of bacteria that are embedded in a matrix of extracellular polymeric substances (EPSs) such as polysaccharides, nucleic acids and proteins [52]. Biofilm production serves as a physical barrier, protecting the population from adverse environmental conditions such as those posed by the immune system and antibiotic administration [53].

Several uFAs have been shown to prevent the formation of *S. aureus* biofilm. Oleic acid has been observed to inhibit primary adhesion during the production of biofilm [54]. At a concentration of 5 µg ml^−1^, roughly 10-fold lower than their MIC, the omega-6 FAs *cis*-4,7,10,13,16,19-docosahexaenoic acid, *cis*-5,8,11,14,17-eicosapentaenoic acid, erucic acid (*cis*-13-docosenoic acid) and gondoic acid (*cis*-11-eicosenoic acid) weakened the virulence of *S. aureus* in a biofilm and *α*-haemolysin-dependent manner [55]. A recent comparison of 27 FAs and their biofilm activity found that 3 C18 uFAs, oleic, petroselinic and vaccenic acids, inhibited biofilm formation at sub-MIC concentrations (100 µg ml^−1^) against *S. aureus* [56]. Additionally, petroselinic acid was shown to inhibit the production of the virulence factors lipase, staphyloxanthin (STX) and *α*-haemolysin. Further transcriptional analysis found that petroselinic acid repressed the expression of *agrA* [56].

Evidence of FA-mediated biofilm dispersion has been observed in diverse bacterial species. For example, *cis*-11-methyl-2-dodecenoic acid produced by *Xanthomonas campestris*, a member of the diffusible signal factor (DSF) family of quorum sensing molecules, promotes biofilm self-dispersion through the activation of endoglucanase, which degrades EPS [57,58]. Similarly, decanoic acid, another DSF member, produced by *Pseudomonas aeruginosa* can induce the dispersal of pre-formed biofilm of several bacterial species including *S. aureus* [59], suggesting that uFAs may act as potent intra- and inter-species biofilm modulatory signalling molecules.

## Mechanism of action of anti-staphylococcal FAs

### Membrane fluidity

In general, membrane fluidity refers to the viscosity of lipids in membrane bilayers and describes the ease of movement of molecules within this membrane environment. The structure of FAs correlates with their antimicrobial activity against *S. aureus*, with numerous investigators reporting that uFAs with *cis*-double bonds are active, whereas saturated and uFAs with *trans*-double bonds have reduced antimicrobial activity [60,61]. uFAs with *cis*-double bonds contain a ‘kink’ in the hydrocarbon tail, which separates adjacent phospholipids and increases fluidity [62]. In contrast, saturated FAs (sFAs) (no double bonds) and uFAs with *trans*-double bonds have a straight hydrocarbon chain, which can pack together tightly in the membrane, decreasing fluidity [63]. For example, *S. aureus* strains with higher levels of saturated membrane lipids have increased membrane rigidity compared with strains with elevated levels of polyunsaturated lipids [64]. Moreover, there is a direct correlation between the degree of unsaturation (i.e. number of *cis*-double bonds) and the potency of uFAs [61,65].

An increase in fluidity can interfere with ATP synthesis as increased movement of electron carriers and proton pumps can impact the ability to generate proton motive force (PMF) [21]. Additionally, leakage of small molecules and ions from the intracellular space can occur, leading to growth inhibition [65]. Cartron *et al*. utilized the fluorescent hydrocarbon 1,6-diphenyl-1,3,5-hexatriene to demonstrate that sapienic acid decreased the polarization index in *S. aureus* cells at sub-lytic and lytic concentrations, indicating a decrease in lipid packing and an increase in membrane fluidity [21].

Additional evidence for uFAs increasing fluidity of bacterial membranes can be inferred from the survival response of *S. aureus* to linoleic acid exposure where genes involved in the synthesis of STX, a carotenoid pigment that reduces membrane fluidity, were significantly upregulated [66]. Interestingly, numerous studies have also shown a direct correlation between the degree of pigmentation and sensitivity of *S. aureus* to uFAs [67,68]. However, the growth in either linoleic or oleic acid has the opposite effect of decreasing membrane fluidity [69,70]. Therefore, initial exposure to uFAs increases fluidity, but long-term exposure to lower concentrations may involve an adaptive response that decreases membrane fluidity.

### Membrane destabilization

If the accumulation of uFAs becomes excessive, the barrier function of the membrane can become compromised. The accumulation of uFAs in the bilayer can cause the formation of transient pores from curvature stress or stable toroidal pores from the bending of the membrane [71,72]. At high concentrations, uFAs can solubilize large fractions of the membrane in a detergent-like fashion where lipids are micellized away from the lipid bilayer [71,72]. Parsons *et al*. used TO-PRO-3 iodide, a membrane-impermeable dye which emits fluorescence upon binding to DNA, to demonstrate that palmitoleic acid treatment disrupted the barrier function of the *S. aureus* membrane. Moreover, the authors identified the release of ATP and low-molecular-weight proteins (<20 kDa) into the supernatant [65]. Rapid release of DNA and ATP was also observed with *S. aureus* cells treated with sapienic acid, and transmission electron microscopy revealed severe morphological defects, including multiple and aberrant positioning of the septa, an effect consistent with the disintegration of the membrane [21].

### Disruption of the electron transport chain

The electron transport chain (ETC) is a series of protein complexes which transfer electrons to generate an electrochemical gradient of protons (PMF) which is utilized by the membrane protein complex, ATP synthase, to generate ATP from ADP and inorganic phosphate (P_i_) [73,74]. The functioning of the ETC is reliant on a stable cell membrane. Therefore, the ability of uFAs to interfere with the ETC relates to their ability to increase membrane fluidity and disrupt the integrity of the lipid bilayer. The disruption of the ETC starves bacteria of ATP, resulting in growth inhibition and eventual cell death [1,62]. Using tetrazolium-based dyes, which are actively reduced by components of the ETC, DeMars *et al*. confirmed that oleic acid treatment resulted in impaired ETC activity in *S. aureus* [70].

### Uncoupling of oxidative phosphorylation

Oxidative phosphorylation refers specifically to the final step of respiration, in which the ATP synthase uses the energy from transporting protons back inside the cell to synthesize ATP [73,74]. ‘Uncoupling’ of oxidative phosphorylation can occur when an external factor facilitates the transport of protons back inside the cell independently of the ATP synthase. This external factor, known as a protonophore, dissipates both membrane potential (ΔΨ) and the proton gradient (ΔpH). FAs are proposed to act as protonophores as they can exist in deprotonated forms, where the carboxyl group is ionized (COO−), and in a protonated form (COOH). In the extracellular space, the ionized FA can be protonated and freely diffuse across the lipid bilayer where it is deprotonated, releasing the H+ ion into the intracellular space. The FA can then flip back across the membrane to transport another proton. This activity provides an alternative route for protons to re-enter the cell, uncoupling the activity of the ETC from ATP synthesis [75,76]. The lipophilic cation tetraphenylphosphonium ion (TPP+) can be used as a marker of ΔΨ. TPP+ accumulates inside the cell under normal conditions as the intracellular space is negatively charged. If a cell becomes depolarized and intracellular space becomes less negatively charged, TPP+ will move into the extracellular space until it reaches an equilibrium [77,78]. Studies using TPP+ have shown that sub-lethal concentrations of sapienic acid lead to the depolarization of *S. aureus* and a reduction in intracellular pH, suggesting that sapienic acid carries protons inside the cell, despite the presence of an intact membrane, and that ΔΨ and ΔpH are disrupted by this activity [21].

### Interference with FA biosynthesis

Bacterial FA biosynthesis is an essential process required to produce phospholipids and is executed by a set of individual enzymes known collectively as the FASII pathway [79]. The final step of FASII that catalyses the rate-limiting chain elongation process is mediated by the enoyl-ACP reductase, FabI. Given the essential nature of this enzyme and the lack of sequence homologue in humans, FabI represents a promising target for anti-staphylococcal therapeutics, with candidates in late-stage clinical trials [80,82]. Intrigued by the antimicrobial properties of unsaturated LCFAs, Zheng *et al*. showed that linoleic acid, palmitoleic acid, oleic acid, linolenic acid and arachidonic acid inhibited FabI, whereas saturated oleic acid (stearic acid) or the methyl ester of linoleic acid lacked FabI inhibition or anti-staphylococcal activity [83]. The authors found that linoleic acid directly bound to FabI, preventing interaction with NADH or NADPH, and also bound to the FabI-NADPH complex, restricting the binding of the substrate. Furthermore, the incorporation of [1-^14^C] acetate into the membrane of *S. aureus* was blocked by linoleic acid, confirming that uFAs can inhibit *de novo* FA biosynthesis in live bacterial cells [83].

Importantly, *S. aureus* has evolved several strategies to resist FabI-targeting antibiotics such as triclosan and AFN-1252. Mutations in FabI which increase resistance to triclosan and AFN-1252 have been identified [84,85], and a significant portion of triclosan-resistant clinical isolates contains mutations in the FabI promoter which result in elevated expression [86]. Furthermore, mutations in *accC* and *accD*, genes involved in FASII initiation, increase resistance to FASII inhibitors via FA auxotrophy, although these mutants require FA supplementation for growth and have decreased virulence in infection models [87,89]. Interestingly, the growth of *S. aureus* in the presence of triclosan and FAs abundant in the human host leads to high-frequency mutations in *fabD* which increase triclosan resistance. Less efficient FabD activity, which uses ACP and malonyl-CoA to synthesize malonyl-ACP, leaves more ACP available for PlsX which can then incorporate exogenous FAs (exoFAs) into phospholipids when FASII is inhibited (FASII bypass) [90]. Similarly, the growth of *S. aureus* with FAs and human serum also increases resistance to FASII inhibitors via FASII bypass, but this occurs independently of mutations. Instead, it was identified that serum protects *S. aureus* from FA-mediated stress, leading to greater retention of ACP for phospholipid biosynthesis from exoFAs [91]. This suggests that the use of FabI inhibitors may be limited against *S. aureus* in certain host environments.

### Lipid peroxidation and auto-oxidation

Lipid peroxidation is the oxidative degradation of FAs where ROS and free radicals can attack uFAs by abstracting a hydrogen atom, adjacent to the double bond, forming a lipid radical. Lipid radicals can react with molecular oxygen to form a lipid peroxyl radical that can also abstract hydrogens from other uFAs, resulting in a chain reaction which continues until it is terminated by antioxidants [92]. Auto-oxidation occurs when PUFAs are exposed to molecular oxygen, which results in the formation of a lipid peroxyl radical without the need for initiation by free radicals [93]. Arachidonic acid is particularly susceptible to these processes as it contains four double bonds and thus more sites for initiation of peroxidation [94]. At each stage of lipid peroxidation and auto-oxidation, numerous antimicrobial components are produced including lipid hydroperoxides, reactive aldehydes (such as malondialdehyde and 4-hydroxynonenal), isoprostanes and *α*,*β*-unsaturated carbonyls. These molecules are highly reactive and, therefore, can have detrimental effects on macromolecules, such as proteins and DNA. Furthermore, they can contribute to the instability of the cell membrane [92]. Lipid peroxidation was initially proposed by Knapp and Melly, as a key antibacterial mechanism of FAs as the addition of catalase, which decomposes hydrogen peroxide and prevents lipid peroxidation initiation, reduced arachidonic acid toxicity towards *S. aureus* [61]. More recently, Beavers *et al*. found that paraquat, which generates ROS to initiate lipid peroxidation, increased arachidonic acid killing of *S. aureus,* whereas manganese, an antioxidant, reduced killing. Further, these authors demonstrated that the lipid peroxidation reaction occurs intracellularly as a membrane-permeable scavenger of the peroxidation product, isolevuglandin, could alleviate arachidonic acid toxicity, whereas an impermeable scavenger had no impact [95]. Overall, these studies indicate that lipid peroxidation of arachidonic acid contributes to anti-staphylococcal activity.

### Cell wall biosynthesis

The exposure of *S. aureus* to uFAs has a significant effect on the transcription of genes involved in cell wall biosynthesis. For example, Kenny *et al*. observed that linoleic acid upregulates the *mraY*, *murD*, *murG* and *murA1* enzymes, involved in the synthesis of peptidoglycan (PGN) precursors, Lipid I and Lipid II. Enzymes which increase the pool of aa required for PGN biosynthesis, such as l-lysine and d-alanine, and several genes involved in wall teichoic acid (WTA) biosynthesis (*tarA*, *tarG* and *tarB*) were also upregulated [66]. Neumann *et al*. observed that sapienic acid treatment upregulates the transcription of *pbp2* and downregulates *pbp4*, enzymes involved in incorporating new PGN precursors into the cell wall and crosslinking the PGN layer, respectively. The transcription of the *dltABCD* operon, which coordinates the addition of a d-alanine residue to WTA and *mprF*, the synthase and flippase of the phospholipid lysyl-phosphatidylglycerol, was also downregulated, suggesting that sapienic acid exposure may decrease cell surface charge [96]. This transcriptional response may be evidence that the integrity of the cell wall is perturbed by FAs. Equally, it could be part of a generalized stress response where altering cell wall properties might prevent FAs from reaching the cell membrane. Recently, our work has demonstrated that the exposure of *S. aureus* to arachidonic acid upregulates the expression of *tcaA*, a key indicator of cell wall stress response via VraRS activation [97]. Interestingly, other studies have observed that linoleic acid upregulates the expression of the major autolysin, *atl*, and downregulates the expression of *cstR*, which inhibits autolysis [98,99]. Correspondingly, *S. aureus* cells grown in the presence of linoleic acid exhibit higher rates of autolysis in a Triton X-100 autolysis assay [66]. Further investigation is needed to determine the effect of uFA membrane insertion on cell wall biosynthesis and the localization of proteins involved in PGN biosynthesis.

### DNA replication

Quinolone antibiotics inhibit bacterial DNA replication by targeting both DNA gyrase, an enzyme that introduces supercoils in DNA ahead of the replication fork to mediate the separation of daughter chromosomes, and topoisomerase IV, an enzyme which removes interlinking between the daughter chromosomes. 2-Hexadecenoic acid (2-HDA) is a synthetic alkynoic FAs which has potent anti-staphylococcal activity and was found to inhibit the supercoiling function of DNA gyrase in a similar fashion to the fluoroquinolone antibiotic, ciprofloxacin [100]. Sanabria-Rios *et al*. hypothesized that this compound binds to the ATPase region of GyrB to inhibit its activity; however, further studies are required for confirmation [100]. It is not known whether natural uFAs can negatively impact DNA replication, but genes involved in DNA replication and repair are upregulated when *S. aureus* is exposed to sapienic acid, which may indicate the occurrence of DNA damage [66,96].

**Fig. 1.**
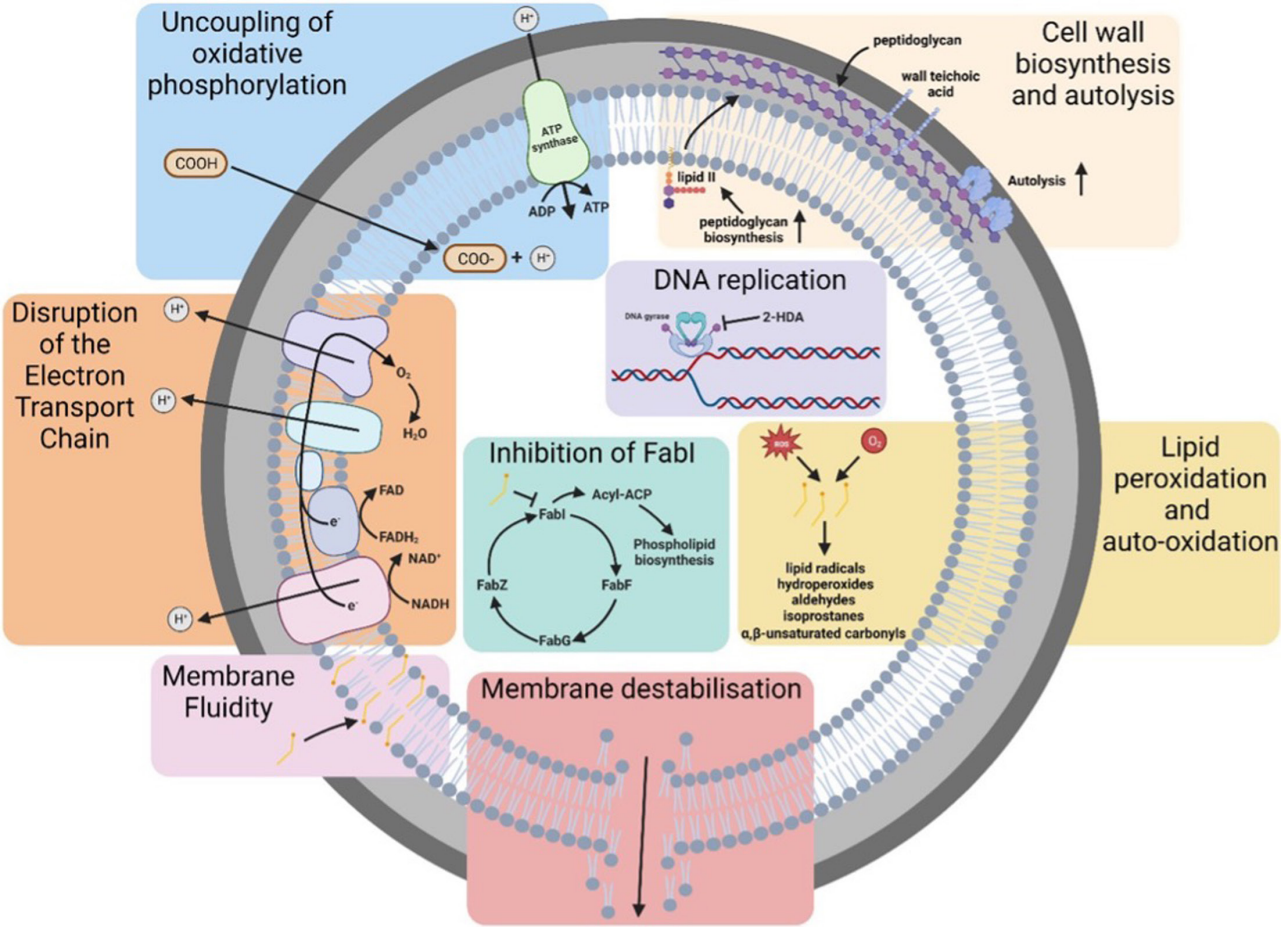
Mechanism of action of anti-staphylococcal FAs.

A schematic of the multiple mechanisms of action of anti-staphylococcal FAs is highlighted in Fig. 1.

## FA resistance mechanisms employed by *S. aureus*

### Hydrophilic surface factors

FAs are highly hydrophobic molecules by virtue of their long hydrocarbon tail. Unsurprisingly, a major defence strategy to thwart FAs is the expression of hydrophilic surface factors, including capsule, WTA and the cell wall anchored iron-regulated surface determinant protein A (IsdA), which reduces the penetration of FAs across the cell envelope.

#### Capsule

The hydrophilic staphylococcal polysaccharide capsule is the outermost component of the cell envelope and is a diverse macromolecular structure with 11 serotypes described in *S. aureus* to date, but with most isolates expressing capsular polysaccharides type 5 (CP5) or type 8 (CP8) [101]. Initial studies focusing on FA resistance mechanisms in *S. aureus* concentrated on capsular expression; Mortensen and Kapral compared the survival of capsulated and non-capsulated *S. aureus* strains in response to FA challenge where a marked decrease in susceptibility was noted for most capsulated strains [102]. However, certain strains were shown to be highly resistant to FAs despite being non-capsulated, which suggested the presence of alternative resistance mechanisms. Whilst genes responsible for capsule biosynthesis are frequently elevated in response to FA challenge [66,96], certain globally successful clones of *S. aureus*, most notably clonal complex (CC) 1 and CC8, are acapsular, due to the presence of mutations (*capE*) or truncations (*capD*) in capsular biosynthetic genes or mutations in the *cap* promoter region [103].

#### Wall teichoic acid

WTA are glycopolymer structures that decorate the cell wall and are comprised of up to 40 ribitol-phosphate subunits that are modified with d-alanine and *N*-acetylglucosamine residues [104]. WTAs play central physiological roles most notably in cell division, protection against host antimicrobial components and as an aid in host colonization [105]. The deletion of *tarO* (*tagO*) which encodes an *N*-acetylglucosamine-phosphate transferase that catalyses the first step of WTA synthesis abolishes WTA production [106]. WTA-deficient mutants display decreased hydrophilicity and enhanced susceptibility to sFAs and uFAs. Loss of WTA resulted in enhanced FA penetration through the cell envelope where they bind more efficiently to the cell membrane [107].

Beavers *et al*. conducted an *in vitro* serial passage experiment to select for arachidonic acid-resistant *S. aureus*, identifying a strain harbouring a K253* mutation in the *lcpA* gene which resulted in a quarter of the catalytic domain being deleted [95]. LcpA is a member of the LytR-CpsA-Psr (LCP) family of proteins, which ligate newly synthesized WTA polymers to PGN [108,109]. Accordingly, mutants lacking the LCP family of enzymes have reduced WTA in the cell wall, which is instead released into the extracellular milieu [110]. It has been shown that the deletion of the entire *lcpA* gene increased resistance to arachidonic acid and demonstrated that treatment of the *lcpA* mutant with subinhibitory tunicamycin, which inhibits TarO, could eliminate the resistant phenotype, highlighting that WTA was required for arachidonic acid resistance. Finally, the authors hypothesized that resistance was dependent on the levels of intracellular ROS, as arachidonic acid kills *S. aureus* through a lipid peroxidation mechanism. Accordingly, they found that the *lcpA* mutant produced less ROS, whilst a *tarO* mutant produced more ROS, correlating with the arachidonic acid sensitivity of these strains [95].

Subsequently, our study identified that mutation of *tcaA*, a gene associated with glycopeptide resistance in *S. aureus*, also increased resistance to arachidonic acid [97]. Moreover, we found that a *tcaA* mutant displayed a similar phenotype to a *lcpA* mutant, where WTA is reduced in the cell wall and released into the supernatant. In this study, we hypothesized that extracellular WTA could sequester or inactivate arachidonic acid in the environment to prevent it from reaching the bacterial cell surface. Accordingly, we demonstrated that the bacterial supernatant of either a *tcaA* or *lcpA* mutant could protect a WT strain from arachidonic acid toxicity. In contrast, the supernatant from a *tarO* mutant had no protective effect. Moreover, the addition of purified WTA extract from a WT strain could also protect from arachidonic acid killing. Together, this data suggests that extracellular WTA in the environment can prevent arachidonic acid from killing *S. aureus*.

#### Iron-regulated surface determinant protein A

Under iron-limiting conditions, IsdA is the most abundant protein expressed on the staphylococcal surface [111]. IsdA is a cell wall-anchored protein that plays key roles in nasal colonization [112,113] and iron acquisition from haem [114]. Clarke *et al*. further investigated the effect of IsdA expression on cell surface physiochemical properties and noted that the growth of several lab strains of *S. aureus* under iron-limited conditions displayed enhanced surface hydrophilicity compared with growth in the presence of iron. The authors confirmed the role of IsdA and specifically the C domain of this protein, to function in mediating the switch in hydrophilic profile and resistance to sFAs and uFAs when grown under iron depletion [22]. Furthermore, by employing a live human skin model, the authors showed that IsdA was required for survival on human skin, suggesting the importance of this protein in mediating resistance to a suite of FAs.

#### SasF

SasF is an *S. aureus* surface protein that is believed to be involved in adhesion and, by virtue of this, colonization. In agreement with this, *sasF* has been shown to be upregulated along with additional members of this family, *sasC* and *sasD*, when *S. aureus* encounters human skin [28]. Prior to this work, Kwiecinski *et al*. established a role for SasF in virulence in a mouse skin model of abscess formation [115]. Furthermore, in the presence of linoleic acid, *sasF* expression was upregulated 32-fold, and a *sasF* mutant was observed to exhibit substantially lower survival rates in linoleic acid-killing assays [66]. A similar result has been shown in *Staphylococcus saprophyticus,* where a *sasF* mutant was shown to be more susceptible to linoleic acid [116]. It was reasoned that SasF may contribute towards FA resistance through altering cell surface hydrophobicity [66], reminiscent of IsdA discussed above. However, a *sasF* mutant generated in the SH1000 background did not display altered hydrophobicity in a hexadecane partitioning assay [66], and thus, SasF may help resist FA toxicity in a manner independent of modifications to surface hydrophobicity.

### Membrane vesicles

Previous studies observed that *S. aureus* grown in the presence of sub-lethal concentrations of FAs survived secondary exposure to a lethal concentration [96,117]. The authors recognized that this adaptation may be the result of an inducible FA stress response system. A subsequent study by Kengmo Tchoupa and Peschel used a linoleic acid analogue to reveal that *S. aureus* prevented FA binding via secretion of a novel factor present in bacterial culture supernatants. The authors discovered that *S. aureus* responded to FA challenge by enhancing the release of membrane vesicles (MVs). MVs were shown to be a general feature released by genetically diverse *S. aureus* strains and showed activity against a broad range of FAs. Although the secretion of MVs increases survival following FA exposure, MVs were shown to contain high levels of lipoproteins, which induce a stronger TLR2-mediated immune response which functions to combat infection [118].

### Detoxification systems

*S. aureus* secretes two enzymes that detoxify FAs into molecules lacking activity: the previously described FA-modifying enzyme (FAME) or Lip2 and more recently discovered oleate hydratase (OhyA).

#### Lip2

Abscess homogenates were shown to possess bactericidal factors that were active against *S. aureus* [119]; however, another unidentified enzyme eliminated this antimicrobial activity, which was subsequently named as FAME [120]. FAME was shown to use a variety of alcohols as a substrate but favoured cholesterol to drive the esterification reaction to form esterified FAs with significantly decreased bactericidal activity [120]. The gene responsible for FAME activity has recently been characterized. Proteomic analysis of *S. aureus* culture supernatant following exposure to subinhibitory concentrations of FAs identified lipases as major determinants of MVs that were previously shown to deactivate FAs. Subsequent genetic mutational analysis confirmed that *lip2* (also referred to as *geh* or *sal2*) was required for protection against FA, with detoxification of FAs requiring the presence of cholesterol [121]. Moreover, an engineered, catalytically inactive *lip2* which showed no lipase activity was also unable to mediate cholesterol-dependent FA resistance. Genomic analysis indicated that *lip2* appeared to be highly conserved across major *S. aureus* lineages; however, the *lip2* sequence displays a conserved integration site for prophages. Interestingly, prophage-mediated disruption of *lip2* was rare in *S. aureus* isolated from the nose, skin and blood, suggesting a role for *lip2*-mediated FA detoxification during colonization and/or infection. Moreover, results from an *in vivo* mouse skin colonization model indicate that *S. aureus* was protected from sapienic acid challenge in a lipase- and cholesterol-dependent manner [121].

#### Oleate hydratases

OhyAs are a large family of enzymes that catalyse the hydration or isomerization of double bonds in uFAs, first isolated from soluble enzyme preparations of *Pseudomonas* spp. [122]. Subramanian *et al*. predicted that the *S. aureus* SA0102 gene encoded an OhyA and subsequently showed using a recombinant version of the protein that it possessed hydratase activity, hydrating only uFAs containing *cis-*9-double bonds, but not FAs containing *trans*-9-double bonds or *cis*-double bonds at positions other than carbon-9 [123]. In this study, palmitoleic acid (C16:1Δ9) was shown to be a substrate for OhyA, and the resulting hydrated product had no antimicrobial activity. Palmitoleic acid completely inhibited the growth of *S. aureus;* however, the growth was subsequently recovered following a 2-h lag period, presumably the duration required for sufficient levels of OhyA to accumulate or the duration needed for OhyA-mediated detoxification of palmitoleic acid. Importantly, the growth of an Δ*ohyA* mutant could not be recovered following the palmitoleic acid challenge. Interestingly, OhyA does portray a degree of substrate specificity as this enzyme could not protect *S. aureus* from sapienic acid (C16:1Δ6) challenge [123]. Importantly, MVs released upon linoleic acid exposure contained high levels of OhyA as well as Lip2 [118], indicating that *S. aureus* coordinates resources to detoxify major antimicrobial FAs.

### Membrane stabilizers

FAs impart membrane perturbation which strongly influences the fluidity and permeability of the bacterial membrane, resulting in growth inhibition [1,72, 95]. To counteract FA-induced membrane damage, *S. aureus* has evolved several membrane-stabilizing strategies outlined below.

#### Type 7 secretion system

The T7SS, also referred to as the ESAT-6 secretion system, is a membrane-embedded secretory system for several important virulence factors [124]. The *S. aureus* T7SS consists of four integral membrane proteins (EsaA, EssA, EssB and EssC) and two cytosolic proteins (EsaB and EsaG) which together orchestrate the secretion of five virulence effectors: EsxA, EsxB, EsxC, EsxD and EsaD [124].

*S. aureus* strains lacking a functional T7SS have been shown to be less virulent in numerous mouse models of infection [69,125, 126]. Intriguingly, FAs have been observed to induce T7SS, a mechanism that is reliant on the incorporation of FAs into the bacterial membrane mediated by the FA scavenging system FakAB [66,69, 126]. Furthermore, recent studies have shown that *S. aureus esxC*, *esxA*, *esxB* and *essC* mutants were more sensitive to linoleic acid and arachidonic acid [127]. Mechanistic dissection of T7SS-mediated FA protection focused on investigations of *S. aureus* lacking EsxC (a representative effector of T7SS) and EssC (the main transporter of T7SS). No significant difference in FA binding was observed between WT and mutant strains, indicating that T7SS components did not participate in the binding or sequestering of FAs [127]. Using the membrane-impermeable fluorescent dye propidium iodide, the authors indicated that mutant strains displayed increased staining compared with the WT, suggesting that an intact T7SS helps to maintain membrane integrity following challenge with FAs. Whole-cell proteomics revealed that T7SS mutants were less able to respond to FA challenge by upregulating proteins associated with hydrolase or oxidoreductase activities. Staining of WT or T7SS mutants with dichlorofluorescin, which reports on the presence of ROS, following treatment with FAs indicated significantly enhanced ROS generation in mutants compared with WT, indicating that T7SS plays a role in mitigating against FA-induced damage [127].

#### Staphyloxanthin

STX is a carotenoid pigment that possesses a long carbon chain skeleton with two terminal rings, located in the cell membrane of *S. aureus* [128]. STX also contains numerous conjugated double bonds that confer antioxidant activity; STX mutants display hypersensitivity to ROS typically found in the phagosome of neutrophils [129]. STX also plays a role in reducing membrane fluidity and functions to stabilize the cell membrane structure, which explains the additional hypersensitivity of STX mutants to antimicrobial FAs [66]. In addition, the incorporation of FAs can alter membrane fluidity, resulting in enhanced expression of STX via activation of the *σ*^B^ stress response [66,130]. Currently, multiple STX inhibitors are being developed [131,132] which may synergize with antimicrobial FAs as a novel combinatorial anti-staphylococcal treatment.

#### Membrane-stabilizing protein A

Membrane-stabilizing protein A (MspA) was discovered through a genome-wide association study that focused on identifying alterations in genetic loci associated with toxin production in *S. aureus* [133]. MspA is a small membrane-bound protein with four membrane-spanning domains. Subsequent studies observed that *mspA* mutants displayed hypersensitivity to innate immune defence molecules, surviving poorly when challenged with oleic acid and human neutrophil peptide-1, and were severely attenuated in superficial and systemic mouse models of infection [134]. Moreover, *mspA* inactivation resulted in significantly lower levels of STX and was more sensitive to membrane-damaging detergents and displayed enhanced membrane permeability [134].

Further work on this protein sought to characterize the function of MspA within the membrane. Through a series of elegant experiments, it was shown that the inactivation of *mspA* triggered over-accumulation of lipoteichoic acid (LTA), which altered autolysin activity and cell separation, causing the cells to inflate in size. Bacterial two-hybrid and three-hybrid assays found that MspA interacted directly with the LTA biosynthesis enzymes UgtP, LtaA and LtaS. Furthermore, MspA was shown to disrupt the interaction between LtaA and LtaS. It was concluded that MspA may act as a ‘dimmer’ switch and helps to maintain the correct physiological levels of LTA within the cell envelope [135]. Taken together, the disruption in the architecture and integrity of the cell envelope of the MspA mutant may render the cell membrane more susceptible to various stresses and explain the increased susceptibility to FAs.

### Efflux pumps and transporters

Synthetic and natural FAs have been shown to inhibit DNA/RNA replication, disturb cell wall and FA biosynthesis, inhibit protein synthesis and disrupt metabolic pathways [136]. As such, numerous efflux pumps that reduce the intracellular accumulation of FAs have been shown to contribute to FA resistance.

#### Tet38

Tet38 is part of the major facilitator superfamily of efflux pumps and has been observed to play a role in antibiotic extrusion [137,138] and to promote internalization and phagolysosome escape of epithelial cells [139]. Expression levels of *tet38* have been shown to be elevated in models of subcutaneous abscess [140] and following exposure to linoleic acid [66]. Moreover, Tet38 as well as the NorB and NorD efflux pumps have been shown to be crucial for the survival of *S. aureus* within abscesses [141,142]. In a subsequent experiment, Truong-Bolduc *et al*. showed that Tet38-expressing strains displayed between a 5- and 8-fold increase in resistance to palmitoleic and undecanoic acid, whilst exposure of *S. aureus* to these FAs was shown to selectively induce the expression of *tet38* transcripts 4-fold [143]. The involvement of Tet38 in FA resistance corresponded well with colonization potential, as a Δ*tet38* mutant exhibited a 5-fold decrease in colonizing potential in a murine skin infection model [143].

#### FarE

FarE was first implicated in *S. aureus* FA resistance by Kenny *et al*., following observations that a putative MMPL efflux pump (SAR2632) was upregulated 2-fold following linoleic acid challenge, and a *SAR2632* mutant displayed decreased survival following linoleic challenge [66]. FarE was later characterized as an efflux pump by the work of Alnaseri *et al*. following a screen to determine the genetic factors involved in *S. aureus* adaptation to growth in the presence of linoleic acid identified a SNP, H121Y, within SAUSA300_2490. Further analysis revealed this gene to encode a TetR family DNA-binding protein designated *farR*, which was accompanied by a divergently transcribed effector gene, SAUSA300_2489, classified as a resistance-nodulation-cell division (RND) efflux transporter, designated *farE* [117].

The *farR* H121Y mutant was found to be a hyperproducer of FarE, and FarR was found to directly regulate the expression of FarE. Additionally, FarR and FarE were demonstrated to be strongly induced in response to linoleic acid, arachidonic acid, sapienic acid, linolenic acid and palmitoleic acid. Mutational analysis revealed a Δ*farE* mutant to be hyper-susceptible to linoleic and arachidonic acids, but not palmitoleic acid. Consistent with an efflux-dependent mechanism of resistance, the inactivation of *farE* resulted in an increased cellular accumulation of radiolabelled [^14^C]-linoleic acid [117]. A follow-up study provided an in-depth characterization of FarR-regulated FarE function. Complementation of a Δ*farER* mutant with either *farE* or *farR* did not restore linoleic acid resistance; however, resistance was restored through complementation of *farER* expressed from their native promoters or ectopic expression of *farE*. The authors also showed that FarR was needed for the expression of *farE* whilst also auto-repressing its own expression. Their experiments also revealed that FarR activates *farE* expression in a manner dependent on a FakA-associated product derived from exoFA metabolism. The authors proposed that this would be to ensure that the efflux of FAs only occurs when the capacity for FA incorporation is exceeded [144].

#### Mnh

A comparative transcriptomic study of *S. aureus* and *Staphylococcus epidermidis* in response to sapienic acid challenge has shown that the *mnhABCDEFG* operon was considerably upregulated [145]. *S. aureus* harbours two multi-subunit Na^+^/H^+^ (Mnh) antiporters which function to maintain cytoplasmic membrane pH, enabling survival under environmental stress [146]. Further analysis confirmed that an in-frame *mnhF* mutant was more susceptible to sapienic acid than WT *S. aureus* [145]. Other studies have reported that a *mnhD* mutant was more sensitive to thrombin-induced platelet microbicidal protein 1 (tPMP-1) [147] and that this operon is also involved in mediating resistance to bile salts and expelling cholic acid [148]. In addition to the *mnh* operon, multiple other cationic transporter systems (CopA, CzrB, OpuD2 and OpuBA) were found to be upregulated in response to sapienic challenge. Perhaps collective upregulation of these transporter systems protects *S. aureus* from the effect of solute leakage, induced through sapienic acid-mediated membrane depolarization [65,145, 149]. Membrane proteins such as FloA and MspA (discussed above) have been shown to provide stability to membrane architecture [150]. Therefore, an alternative hypothesis is that Mnh may provide some mechanical stability/strengthening to the bacterial membrane, thus protecting it from the membrane-damaging effects of FAs.

### FA metabolism

*S. aureus* can produce sFAs through the action of the resident FASII system. However, it can also scavenge exoFAs from the host environment through the action of the FA kinase (FAK) system [151,152]. The FAK system is comprised of the FAK FakA which works in conjunction with the FA-binding proteins FakB1 and FakB2 [152]. In *S. aureus*, FakB1 is used to remove saturated exoFAs, and FakB2 is used to remove unsaturated exoFAs from the bacterial membrane [153]. Once removed, FakA proceeds to phosphorylate the carboxyl head of the exoFA to create an acyl phosphate which can then be fed into the phospholipid pathway [152]. Non-*S. aureus* FAs can be incorporated into the bacterial membrane impacting lipid packaging and composition, leading to an alteration in the fluidity of the membrane [70].

It stands to reason that *S. aureus* utilizes the FAK system to ensure the passive accumulation of FAs into the membrane does not result in a catastrophic loss of membrane barrier function. However, proving this experimentally is challenging. In support of the FAK system, being an FA resistance determinant is the observation that other key FA resistance strategies are intrinsically linked to FAK metabolites. For example, we have discussed above how exoFA breakdown products interact with FarR to ensure that *farE* expression only occurs once the metabolic capacity for exoFA incorporation is exceeded [144]. Similarly, it has been observed that the T7SS is induced in response to FA scavenging by FAK [144]. Paradoxically, the deletion of *fakA* or a dual deletion of *fakB1* and *fakB2* leads to enhanced resistance to FAs [154]. Specifically, Δ*fakA* mutants were found to be more resistant to linoleic and oleic acid, which was phenocopied by the Δ*fakB1/B2* double mutant [154]. Enhanced resistance in the absence of FakA has been attributed to elevated basal levels of *farE* expression [144], as well as pleiotropic effects on *S. aureus* transcriptional signalling. Specifically, USA300 Δ*fakA* is associated with reduced transcription of the SaeRS two-component system which contributes to defective Hla production [155]. The expression of *sigB* was also increased roughly 8-fold in the Δ*fakA* mutant, and its resistance profile could be reduced to an intermediary phenotype when *σ*^B^ was deleted [154]. *σ*^B^ has previously been implicated in FA resistance, where a Δ*sigB* mutant was more sensitive to linoleic acid challenge compared with the WT strain [66].

This contradiction is further exemplified when looking at mouse models of infection using WT and *fakA* mutants. Whilst the inactivation of *fakA* does not affect *S. aureus* proliferation [156], *fakA* mutants are outcompeted [157] or found in lower abundance [69], compared with the WT in systemic mouse infections. However, *fakA* mutants provoke larger lesions and contribute to increased dermonecrosis than the WT during murine skin infection [158]. Therefore, whether exoFA incorporation by *S. aureus* functions as an antimicrobial FA resistance mechanism remains unclear.

*S. aureus* is also capable of metabolizing FAs through *β*-oxidation. In this pathway, FadD activates LCFAs through conversion into acyl-CoA. FadE then generates a trans-enoyl acyl-CoA intermediate, and FabB sequentially converts this into hydroxy acyl-CoA and then *β*-keto acyl-CoA. Finally, FadA cleaves *β*-keto acyl-CoA to release acetyl-CoA, and the shortened carbon chain is combined with CoA-SH to allow for additional cycles of *β*-oxidation to occur [159,160]. The genes for this pathway are in a single locus in *S. aureus*, *fadXDEBA*, where *fadX* is an alternative acyl-CoA transferase to *fadD* which can activate SCFAs. The deletion of the entire *fadXDEBA* operon (USA300∆*fad*) decreases viability in the presence of palmitic acid [160]. Whilst sFAs are non-toxic towards *S. aureus*, C16 acyl-phosphate formed from palmitic acid is a poor substrate for phospholipid biosynthesis. These chains are instead elongated by FabF for efficient utilization by the PlsY acyltransferase [161]. Interestingly, USA300∆*fad* has increased incorporation of C16 into phosphatidylglycerol upon exposure to palmitic acid and concurrently does not produce palmitoyl-CoA, the first metabolite in *β*-oxidation [160]. Combined, these data suggest that *fadXDEBA* protects *S. aureus* from a metabolic bottleneck caused by palmitic acid incorporation into PGN by drawing it away from phospholipid biosynthesis.

**Fig. 2.**
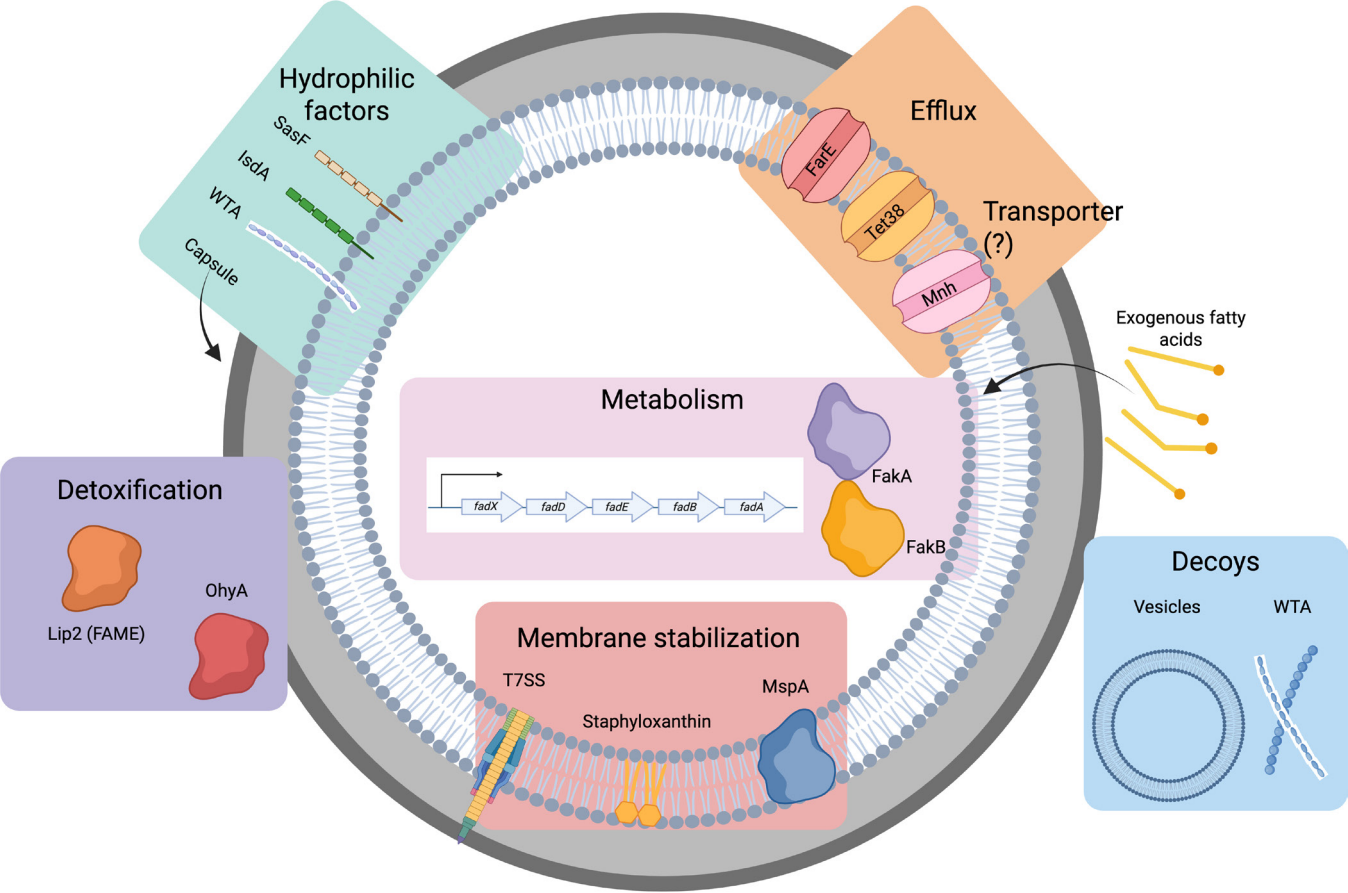
Strategies employed by *S. aureus* to resist FAs antimicrobial activity.

An illustration of the FA resistance strategies utilized by *S. aureus* is provided in Fig. 2.

## Therapeutic application

FAs are attracting attention due to their clinical potential for the treatment of *S. aureus* infections. In addition to being extremely potent, they are also broad-spectrum and have activity against MDR *S. aureus*, and their chemical structures are amenable to modifications aimed at enhancing activity. Furthermore, they are generally considered non-toxic and harbour additional anti-inflammatory properties that could contribute to infection resolution [2,162].

### FAs for decolonization and treatment of skin infections

Whilst some studies have suggested the use of FAs to treat systemic infections [22,163], the most likely prospect for exploiting FAs therapeutically is as a topical agent. Currently, topical treatment of superficial *S. aureus* infections and nasal decolonization prior to hospital admission relies on the use of fusidic acid, mupirocin, clindamycin or retapamulin ointments [8]. However, declining clinical efficacy due to the increasing prevalence of MDR *S. aureus* strains highlights the need for alternative therapies [164,165].

Multiple studies have observed success using FAs as therapeutic *S. aureus* decolonization agents. Lacy and Lord performed *S. aureus* survival assays on human skin with and without the addition of neat linoleic acid. In the untreated patient group, a high percentage of the initial inoculum was recovered after 6 h. Conversely, for the linoleic acid-treated group, <0.01% of the starting inoculum was recovered in the same time period [166]. Other studies investigated the use of lauric acid monoester (LAM), observing *in vitro* effectiveness against methicillin-susceptible *S. aureus* (MSSA) and MRSA isolates and MIC_90_ values of <4 µg ml^−1^ versus 0.25 µg ml^−1^ for mupirocin. An *in vivo* murine model of MRSA nasopharyngeal colonization indicated that LAM formulations were more effective than mupirocin in eradicating *S. aureus* carriage following 3 days of treatment [167].

FA and FA formulations have shown effectiveness in the treatment of murine skin infections. Packaging of oleic acid into liposomes (LipoOA) as a novel delivery system displayed bactericidal activity at concentrations of 12.5 µg ml^−1^ against MRSA252, 12-fold more effective than free oleic acid [168]. The increased potency was suggested to be the result of the increased fusion of oleic acid into the bacterial membrane. The therapeutic efficacy of LipoOA was further evaluated in a mouse skin infection model; MRSA252 (1×10^7^ c.f.u.) was injected into the skin alongside either control, FA-free liposomes or LipoOA. Following 24 h, skin lesions formed in the control liposome group but not the LipoOA. Further homogenization of skin tissues showed that the amount of MRSA252 remaining was 500-fold less in the LipoOA compared with the bare liposome treatment group [168]. Future studies evaluating the therapeutic effect once skin lesions are formed are required. The ability of oleic acid to resolve mouse skin infections has further been demonstrated by Chen *et al.* when directly administered in the same location as the subcutaneous injection of *S. aureus*. Oleic acid treatment significantly attenuated skin lesion formation and alleviated the skin damage caused by the infecting *S. aureus* strain [169].

Sapienic acid has also shown promise in the treatment of MSSA disease in both a murine model of systemic infection and AD where either injection or topical application of sapienic acid 2–4 h after infection resulted in a significant reduction in the *S. aureus* burden [22].

### Synthetic FAs

#### Furan FAs

Recently, there has been a focus on understanding and manipulating the chemical structures of FAs such to improve their potency and/or spectrum of activity. Furan FAs, characterized by a furan ring and an unbranched 9, 11 or 13 carbon chain FA, are important human biologics displaying antioxidant, antithrombotic, anti-inflammatory and cardioprotective properties [170]. Using a microbial biotransformation approach, Ellamar *et al.* synthesized a novel, biologically active furan FA [7,10-epoxyoctadeca-7,9-dienoic acid (7,10-EODA)] [170]. 7,10-EODA exhibited an MIC range of 125–250 µg ml^−1^ against a cohort of MSSA and MRSA strains and prevented the production of haemolysins, autolysins and proteases at subinhibitory concentrations [171]. Furthermore, 7,10-EODA synergized with *β*-lactams, facilitating increased antibiotic binding, resulting in fractional inhibitory concentration indexes below the 0.5 threshold against clinical MRSA isolates [172].

#### Acetylenic acids

Acetylenic FAs are typically found in plants and used as a component of their natural defences against micro-organisms. This class of FAs contains a highly peculiar C≡C and has been of interest to medicinal chemists due to their antimicrobial activity towards nosocomial pathogens. Synthetic 2-alkynoic FAs, such as 2-HDA, have received attention for their impressive antimicrobial activity. Structure–activity relationship studies of 2-HDA demonstrated that the C≡C bond at position 2 in hexadecenoic acid is pivotal to its antimicrobial potency [173] and that the antibacterial activity of hexadecenoic acid decreases as the triple bond is moved further away from the terminal carboxyl group [100]. 2-HDA exhibits activity against a range of problematic bacterial species including *S. aureus* with MIC values of 15.6 µg ml^−1^ [173]. 2-HDA retained activity against MRSA isolates resistant to ciprofloxacin, which the authors linked to its capacity to inhibit DNA gyrase [100].

#### *α*-Methoxylated FAs

The *α*-methoxylated FAs are unusual 14–28 carbon FAs that possess a methoxy branch and are recovered from a number of different Caribbean sponge species [174]. Carballeira *et al.* reported the complete synthesis of (±)-2-methoxy-6-hexadecenoic acid which displayed higher potency than the naturally derived 6-hexadecenoic (sapienic acid) [175]. The authors also synthesized the synthetic acetylenic acid derivatives of both (±)-2-methoxy-6-hexadecenoic acid and (6*Z*)-(±)-2-methoxy-6-octadecenoic acid (2M,6-ODA); the addition of a C≡C bond in (±)-2-methoxy-6-hexadecenoic acid did not enhance potency. However, the acetylenic derivative of 2M,6-ODA was the most potent methoxylated FA tested with an MIC range of 31–125 µg ml^−1^ against *S. aureus*.

### FA-AMP conjugates

AMPs are generally short (10–50 aa) with a net positive charge (+2 to +9) and a high proportion of hydrophobic residues (>30%) that exhibit broad antimicrobial activity [176,177]. Upon contact with target membranes, AMPs adopt an amphipathic structure, and the hydrophobic residues insert in the bilayer to form transient pores. In addition, the peptide can aggregate on the surface to induce a detergent-like disintegration. Both mechanisms disrupt essential membrane processes and lead to leakage of intracellular contents [176,178].

AMPs are of significant interest for future therapeutic development; however, challenges related to insufficient potency and lack of activity and stability under physiological conditions are important limitations. Lipidation, the chemical covalent attachment of FA chains to AMPs, shows promise in circumventing obstacles related to the therapeutic use of AMPs. Lipidation has been shown to enhance bioactivity; for example, conjugation of C-12 FAs to a short peptide sequence derived from human cathepsin G (N-terminal sequence 117–136) increased antimicrobial activity 16–32-fold compared with non-lipidated AMPs against a panel of *S. aureus* isolates [179]. To investigate the importance of FA acyl chain length on the antimicrobial activity of FA-AMP conjugates, Mak *et al.* used the human cathepsin G N-terminal sequence 117–136 and modified this sequence with FAs ranging from C-2 to C-18 hydrocarbons. The authors observed an increase in *α*-helical formation of the peptides up to a chain length of C-12, but no change in structure was observed in peptides containing C-14 to C-18. This data correlated with the ability of FA-AMP conjugates to lyse large unilamellar membranes and display antimicrobial activity against MRSA and vancomycin-intermediate isolates of *S. aureus* [179]. Chu-Kung *et al.* conjugated FAs ranging from C-12 to C-20 in length to a synthetic leucine-lysine AMP and measured the critical micelle concentration of the peptides using pyrene solubilization and tensiometry [180,181]. The authors identified higher aggregation of the peptides into micelles as hydrocarbon chain length increased. Importantly, if the peptides aggregated at a concentration below the minimum bactericidal concentration, no antimicrobial activity was observed, highlighting that self-assembly at high hydrophobicity inhibits AMP activity.

Lipidation can also improve the antimicrobial activity of AMPs when tested under conditions aiming to mimic physiological conditions. Human serum albumin can sequester AMPs, diminishing activity due to its negative charge; Grimsey *et al.* observed that the addition of C-12 and C-14 FAs to synthetic AMP domains increased the activity against MRSA in the presence of 25% human serum between 4- and 8-fold compared with the parent peptide [182].

Physiological salt concentrations can also be inhibitory for AMP activity as they interfere with electrostatic interactions between the peptide and the bacterial membrane. Zhong *et al.* conjugated C-10 and C-12 FAs to the N-terminus of anoplin-D4,7 and found that antimicrobial activity was much higher against *Escherichia coli* and *S. aureus* in the presence of physiological concentrations of NaCl, KCl, MgCl_2_, ZnCl_2_ and FeCl_3_, compared with the parent peptide [183]. In support of these observations, Krishnakumari and Nagaraj demonstrated that N-terminal palmitoylation of the C-terminal segment of bovine-*β*-defensin-2 allowed the peptide to maintain activity against *E. coli* and *S. aureus* in the presence of 150 mM NaCl. In contrast, the parent peptide has no activity under these conditions. The authors proposed that the FA chain acts as an anchor to solidify electrostatic contact between the AMP and bacterial membrane and allow for pore formation in the presence of physiological salt concentrations [184].

### Antibiotic potentiation

Lipophilic side chain modification of the glycopeptide antibiotic, vancomycin, can improve potency as the modified antibiotic can anchor to the membrane and induce secondary membrane-damaging effects [185]. This antibiotic potentiation served as the rationale for the work of Sidders *et al.*, who proposed that partnering vancomycin with cell membrane-active agents like FAs could improve activity [186]. Palmitoleic acid, linoleic acid and rhamnolipids (RLs) (glycolipids consisting of l-rhamnose and *β*-hydroxy FA moieties) were observed to potentiate vancomycin killing but not other cell wall targeting antibiotics such as *β*-lactams and fosfomycin. Further molecular dissection focussing on vancomycin/palmitoleic acid found that the synergistic activity was due to the accumulation of membrane-bound cell wall precursors. This accumulation led to the generation of large fluid patches within the membrane which caused protein delocalization and a loss of membrane integrity [186]. FA-vancomycin synergy has similarly been noted with omega-3 FAs and has proved to be an effective combination in alleviating osteomyelitis caused by *S. aureus* in rats [187].

Aminoglycosides are intracellularly active antibiotics that rely on PMF to enter the bacterial cell [188]. As a facultative anaerobe, *S. aureus* can effectively switch off PMF and reduce aminoglycoside entry by switching between the energy-yielding metabolic pathways of respiration and fermentation [189,190]. Consequently, higher concentrations of aminoglycosides that can cause significant adverse reactions to the host are required to eradicate fermentative *S. aureus* [191]. Recently, Radlinski *et al.* demonstrated that GML and RLs acted as potent tobramycin adjuvants and could restore tobramycin susceptibility to highly antibiotic-resistant *S. aureus* persister cells [189]. Interestingly, the RL challenge but not GML resulted in enhanced aminoglycoside uptake due to RL-mediated modifications of cell surface charge, membrane fluidity and small molecule permeability. Moreover, treatment with RL resulted in perturbation of membrane-associated cell-division machinery, whereas GML caused membrane clumping suggestive of significant membrane destabilization [189].

Similar antibiotic potentiation observations have been noted with arachidonic acid partnered with gentamicin, resulting in a substantial reduction in *S. aureus* viability compared with individual gentamicin treatment. This was suggested to be due to arachidonic acid increasing membrane fluidity as palmitic acid, which is fully saturated and does not increase membrane fluidity, did not synergize with gentamicin [192]. A screen of 15 sFAs and 15 uFAs for synergy with tobramycin found myristoleic acid-tobramycin to reduce bacterial viability by >4 log compared with tobramycin treatment alone against MSSA and MRSA isolates. The aminoglycoside potentiation effect of myristoleic acid was also demonstrated with tobramycin, kanamycin, gentamicin and streptomycin [193].

## Conclusions

FA and FA-combinational therapies represent promising anti-staphylococcal treatment options, whilst FA monotherapy displays additional anti-infective activity by blocking the expression of key *S. aureus* virulence factors central to disease.

The site of action of FAs is primarily the bacterial membrane. Studies have shown that FA exposure results in severe damage to membrane physiology, altering membrane fluidity, increasing membrane permeability and disrupting the ETC. Further dissection of FA mode of action identified FabI, a core enzyme of staphylococcal FA biosynthesis as a target. Moreover, lipid peroxidation of specific FAs such as arachidonic acid was demonstrated to be a key feature of antimicrobial activity. *S. aureus* transcriptomic analysis following exposure to FAs revealed significant alteration in genes involved in cell wall biosynthesis, hinting at a potential role of FAs in disrupting cell wall synthesis. Resistance to FAs exists in the form of structural barriers, surface determinants that repel the hydrophobic FAs, secreted MVs and detoxification enzymes that inactivate FAs. In addition, integral membrane stabilizers defend against FA membrane insertion and disruption, whilst increasing evidence indicates that efflux pumps play a key role in staphylococcal FA resistance.

Whilst the use of FAs as therapeutic agents is attractive, there are several significant gaps in the literature that impede future development. Given the high concentrations of FAs required to eliminate bacterial pathogens including *S. aureus*, further research on FA toxicity to human cells and organ systems is essential. The activity of FAs and FA combinations in clinically relevant environments, for example, in the presence of human serum or under physiological salt conditions, is relatively unknown but does represent significant limitations for the clinical implementation of host-derived AMP-based therapeutics [194]. Experimental evidence informing on the types of infection that are best suited for FA-based therapies is lacking; most animal infection studies focus on topical FA infection control applications and provide no data on the critical pharmacokinetic and pharmacodynamic properties of FAs. Lastly, little information is available on the frequency of resistance to FAs and whether resistance determinants can be mobilized and rapidly exchanged.

Research in developing more potent FAs through profiling molecular-level membrane interactions may help predict potency and synergistic potential, promoting the rational design of future therapeutics. FA-AMP conjugates and improved FA delivery systems have the potential to improve potency and stability and decrease cytotoxicity. Continued development in antibiotic and FA mode of action discoveries has the potential to identify future FA-based antibiotic adjuvants that will help preserve and improve current therapies aimed at combating MDR *S. aureus* infections.

## Abbreviations

AD: atopic dermatitis
AMP: antimicrobial peptide
CC: clonal complex
COX: cyclooxygenase
DSF: diffusible signal factor
7,10-EODA: 7,10-epoxyoctadeca-7,9-dienoic acid
EPS: extracellular polymeric substance
ETC: electron transport chain
exoFA: exogenous FA
FA: fatty acid
FAK: FA kinase
FAME: FA-modifying enzyme
GML: glycerol monolaurate
2-HDA: 2-hexadecenoic acid
HFD-P: polyunsaturated high-fat diet
HFD-S: saturated high-fat diet
IsdA: iron-regulated surface determinant protein A
LAM: lauric acid monoester
LCFAs: long-chain FAs
LCP: LytR-CpsA-Psr
LTA: lipoteichoic acid
MDR: multidrug resistant
2M,6-ODA: (6*Z*)-(±)-2-methoxy-6-octadecenoic acid
MRSA: methicillin-resistant *S. aureus*
MspA: membrane-stabilizing protein A
MSSA: methicillin-susceptible *S. aureus*
MVs: membrane vesicles
OhyA: oleate hydratase
PG: phosphatidylglycerol
PGN: peptidoglycan
PMF: proton motive force
PUFA: polyunsaturated FA
RL: rhamnolipid
ROS: reactive oxygen species
SCFA: short-chain FA
sFAs: saturated FAs
STX: staphyloxanthin
TPP+: tetraphenylphosphonium ion
T7SS: type 7 secretion system
uFAs: unsaturated FAs
WTA: wall teichoic acid
